# The Etiology of Puerperal Cystitis

**Published:** 1880-04

**Authors:** 

**Affiliations:** Halle, A. S.


### THE ETIOLOGY OF PUERPERAL CYSTITIS.

BY SWARTZ, OF HALLE, A. S.

THE author supports the view that cystitis in lying-in women is a disease which arises by infection. Injuries received during parturition cannot, any more than the mechanical irritation of the bladder and urethra by repeated introduction of the catheter during labor, set up cystitis. Retention of urine must also be rejected as a cause; for spontaneous decomposition of retained urine never occurs. The causes of puerperal cystitis, are: (1)

the introduction of phlogenic material directly into the bladder by the catheter, and (2) the spontaneous extension to the vesical mucous membrane of inflammatory processes in its neighborhood. A collection of thirty-two cases of this disease out of about 1100 lying-in women in the Midwifery Clinique at Halle, A. S., in the years 1868—75, shows that in by far the majority of cases, the disease arose from the introduction of infective material directly into the bladder by the catheter. Among these twenty-two cases of puerperal cystitis (twenty of which were simple cystitis, and twelve complicated with pyelitis) only two arose from the extension of inflammation in the neighborhood; the remaining thirty (in twenty-one quite certainly, in nine with the greatest probability) as result of the introduction of phlogenic material by the catheter.

This cystitis does not especially affect those lying-in women who have had difficult labors, nor those who suffer from inflammatory affections in the neighborhood of the urethra and bladder; but such as have been catheterized. Antiseptic measures (especially the entrance of air into the bladder is to be avoided, to do which the catheter should be introduced into the bladder filled with carbolic solution) securely guard against the introduction of infective material. Since 1875, these precautions have been employed in the Halle Midwifery Clinique, and puerperal cystitis, arising from catheterization, has become very rare. It must be mentioned that the author accepts the view that the lochial secretion, under entirely normal conditions, may contain the injurious material.—*Centralblatt für Gynäkologie.*

